# Visible DNA microarray and loop-mediated isothermal amplification (LAMP) for the identification of *Cryptococcus* species recovered from culture medium and cerebrospinal fluid of patients with meningitis

**DOI:** 10.1590/1414-431X20209056

**Published:** 2020-10-09

**Authors:** P. Stivanelli, C.A. Tararam, P. Trabasso, L.O. Levy, M.S.C. Melhem, A.Z. Schreiber, M.L. Moretti

**Affiliations:** 1 Departamento de Medicina Interna, Faculdade de Ciências Médicas, Universidade Estadual de Campinas, Campinas, SP, Brasil; 2 Departamento de Patologia Clínica, Faculdade de Ciências Médicas, Universidade Estadual de Campinas, Campinas, SP, Brasil; 3 Instituto Adolfo Lutz, São Paulo, SP, Brasil; 4 Faculdade de Medicina, Universidade Federal de Mato Grosso do Sul, Campo Grande, MS, Brasil; 5 Centro de Pesquisa em Obesidade e Comorbidades (CEPIDI), Universidade Estadual de Campinas, Campinas, SP, Brasil

**Keywords:** Cryptococcus, Microarray, LAMP, Molecular diagnosis, Cryptococcal meningitis

## Abstract

Cryptococcal meningitis affects normal hosts and immunocompromised patients exhibiting high mortality rates. The objective of this study was to design two molecular assays, visible microarray platforms and loop-mediated isothermal amplification (LAMP), to identify *Cryptococcus* spp. and the species *neoformans* and *gattii* from the cerebral spinal fluid (CSF). To identify *Cryptococcus* and the two species, we designed two microarrays DNA platforms based on the internal transcribed spacer (ITS) region and *CAP59* gene and LAMP assays specific for *Cryptococcus* species. The assays were tested using CSF from patients with cryptococcal meningitis. CSF from patients with cryptococcal meningitis was cultured in Sabouraud culture medium, and the *Cryptococcus* spp. grown in the culture medium were also tested for LAMP and microarray platforms. The results were compared to DNA sequencing of the same genetic regions. A total of 133 CSF samples were studied. Eleven CSFs were positive for *Cryptococcus* (9 *C. neoformans* and 2 *C. gattii*), 15 were positive for bacteria, and 107 were negative. The *CAP59* platform correctly identified 73% of the CSF samples, while the ITS platform identified 45.5%. *CAP59* platform correctly identified 100% of the *Cryptococcus* isolates, and ITS platform identified 70%. The two sets of LAMP primers correctly identified 100% of the *Cryptococcus* isolates. However, for CSF samples, the amplification occurred only in 55.5% of *C. neoformans.* The methodologies were reliable in the identification of *Cryptococcus* species, mainly for isolates from culture medium, and they might be applied as adjunctive tests to identify *Cryptococcus* species.

## Introduction

Cryptococcosis is the leading systemic fungal infection worldwide with an estimated number of cases per year close to 1 million (1). In Brazil, cryptococcosis is associated with the AIDS epidemic when 40,000 new AIDS cases were being identified annually (2), coupled with 1,000 to 2,500 new cases of cryptococcal meningitis leading to an approximately 40% mortality rate (1,3). The cryptococcal meningitis caused by *Cryptococcus neoformans* and *Cryptococcus gattii* are clinically indistinguishable, however, some authors suggest that meningitis due to *C. gattii* appears to be more aggressive than that caused by *C. neoformans,* and may require extended periods of treatment (4,5).

The microbiological identification by automated systems and phenotypic methods applied in the differentiation of *Cryptococcus* species requires confirmation by molecular methods. Recently, matrix-assisted laser desorption ionization-time of flight mass spectrometry (MALDI-TOF MS) has identified correctly close to 100% of *Cryptococcus* species (6); however, this technology is available in few hospitals in Brazil. Immunoassays for the diagnosis of cryptococcosis have been applied in clinical practice to detect cryptococcal capsular antigen in body fluids, such as the *Cryptococcus* antigen lateral flow assay (CrAg LFA). The CrAg LFA assay identifies the four main serotypes of *Cryptococcus* (A, B, C, and D) (7), but not to species level. Cerebral lesions and hydrocephalus are more common in infections caused by *C. gattii* (8,9). In these cases, the accurate species identification would be useful in guiding the appropriate therapy scheme. The CrAg LFA assay is produced by only one biotech company (IMMY^®^, USA), and has not yet been approved by the Brazilian Health Regulatory Agency (Anvisa), which is an autarchy of the Ministry of Health that regulates health products in the country. Because the CrAg LFA assay is not available here, other techniques, including molecular ones, are necessary to improve the diagnosis of cryptococcosis.

A reliable identification of *Cryptococcus* species may facilitate the proper antifungal treatment and improve clinical and epidemiological surveillance strategies. Several studies have been published on the application of molecular techniques for the identification and diagnosis of *Cryptococcus*, but there is still a need for new studies and technologies to implement the diagnosis of this mycosis (10–13).

Molecular techniques have contributed significantly to improve the diagnosis of fungal diseases. DNA sequencing has been an important complementary method to classical mycological identification of fungal pathogens, but it is time-consuming and expensive. Thus, there is a need for simpler molecular diagnostic methodologies with similar specificity. DNA microarray platforms have been developed to identify a diversity of microorganisms such as viruses (14), bacteria (15), and fungi (16,17), simultaneously or individually, with good sensitivity and specificity. Due to the low stability of most fluorescent dyes and the expensive scanning equipment used in this technique to visualize the result, our research group developed a more accessible DNA microarray system for identification of fungi at the genus and species level, the result of which is visualized with the naked eye (18).

Loop-mediated isothermal amplification (LAMP) has been used as a molecular diagnostic methodology based on the principle of isothermal amplification of simple nucleic acid. This technique has shown to be fast, specific, and economical. The assay has been validated by different clinical studies presenting a similar performance as the conventional PCR (19,20), and it has been applied for the detection of several pathogenic microorganisms, including viruses (20,21), bacteria (22), protozoa (23), and fungi (24–29).

Therefore, the objective of this study was to develop two assays based on molecular techniques, visible DNA microarrays and LAMP, to identify the genus *Cryptococcus* and the species *neoformans* and *gattii* directly from clinical samples of cerebral spinal fluid (CSF) and from isolates of *Cryptococcus* grown on culture plates.

## Material and Methods

### Study design

The study was performed at the Clinical Hospital of the University of Campinas, Sao Paulo, Brazil, which is the referral hospital for tertiary care for more than 3 million inhabitants. All the laboratory assays were performed at the Molecular Epidemiology and Infectious Diseases Laboratory (LEMDI) of the Faculty of Medical Sciences, University of Campinas. This study was approved by the Institutional Ethical Committee (No. CAAE: 54943116000005404). We tested *Cryptococcus* isolates grown on culture plates after CSF cultivation and *Cryptococcus* spp. from the culture collection of Instituto Adolfo Lutz (IAL), which is the reference laboratory for infectious diseases for the State of São Paulo and Federal governments. LAMP and two visible DNA microarray platforms were tested.

### Clinical samples

From August 2016 to October 2018, we studied samples of CSF from patients hospitalized at the Clinical Hospital. The microbiology laboratory of the Clinical Pathology Department previously analyzed the CSF samples. An aliquot was stored at 4°C as counterproof for further studies. For our study, we tested the CSFs that were positive for bacteria, *Cryptococcus* spp., and the ones that were negative for bacteria, mycobacteria, and fungi. The microbiological identification was performed by the automated system Vitek^®^ 2 (Lab Equipment bioMérieux Inc., USA) or BD Phoenix (Becton Dickinson, USA). The results of fungal identification obtained with the automated systems were compared with the ones from DNA microarray, LAMP, and DNA sequencing. All CSF samples were submitted to DNA extraction (see methods) including the ones with a positive microbiological identification for fungi, bacteria or that cultured negative for pathogens. DNA sequencing, LAMP, and DNA microarray were performed for all CSF samples in which DNA extraction resulted in a positive amplification in the PCR reaction. The molecular tests were performed after all the CSF standard analyses and microbiological and biochemical tests were completed. Our results were not reported to the assistant physician and they were not used for therapeutic proposes.

For the purpose of this study, a loop of all CSF samples was cultivated in agar Sabouraud. The yeasts recovered from the culture plates were then stored in sterile water at 4°C and in 10% glycerol at −80°C.

### Yeast strains

Eleven *Cryptococcus* spp. strains from CSF samples and 30 clinical and environmental isolates identified as *C. gattii*, generously donated by the Mycology Division of the IAL (Table 1), were included to develop the molecular assays of *Cryptococcus* species identification. The DNA samples of *C. neoformans* genotypes VNI to VNIII and *C. gattii* VGI to VGIII were kindly provided by the Oswaldo Cruz Foundation (FIOCRUZ) for LAMP assays.

**Table 1:** Cerebral spinal fluid (CSF) samples used in this study according to sample number and microbiological identification.

CSF samples	Sample Number	Fungal identification in CSF by Vitek^^®^^ 2 or BD Phoenix	Patient case number
Group A (n=11)	P232	*Cryptococcus* spp.	Case 1
	P260	*Cryptococcus neoformans*	Case 2
	P261	*C. neoformans*	Case 2
	P272	Negative	Case 3
	P273	*C. neoformans*	Case 4
	P274	*C. neoformans*	Case 4
	P275	*C. neoformans*	Case 5
	P276	*Cryptococcus* spp.	Case 6
	P283	*C. neoformans*	Case 7
	P284	*C. neoformans*	Case 8
	P285	*Cryptococcus gattii*	Case 9
Group B (n=15)	P63	*Staphylococcus haemolyticus and Staphylococcus epidermidis*	
	P113	*Pseudomonas aeruginosa*	
	P228	*Enterobacter cloacae*	
	P240	*Staphylococcus aureus*	
	P257	*P. aeruginosa*	
	P259	*P. aeruginosa*	
	P262	*P. aeruginosa*	
	P263	*Streptococcus pneumoniae*	
	P264	*E. cloacae*	
	P265	*Staphylococcus caprae*	
	P266	*S. caprae*	
	P267	*Haemophilus influenzae*	
	P268	*S. pneumoniae*	
	P269	*Pantoea aerogenes*	
	P270	*S. pneumoniae*	
Group C (n=107)		Negative	
IAL samples (n=30)		*C. gattii*	

Group A: 11 CSF samples from 9 patients that cultured positive for *Cryptococcus*; Group B: 15 CSF samples from patients with bacterial meningitis; Group C: 107 CSF samples that cultured negative for fungi, bacteria, and mycobacteria. IAL: Instituto Adolfo Lutz.

**Figure 1:**
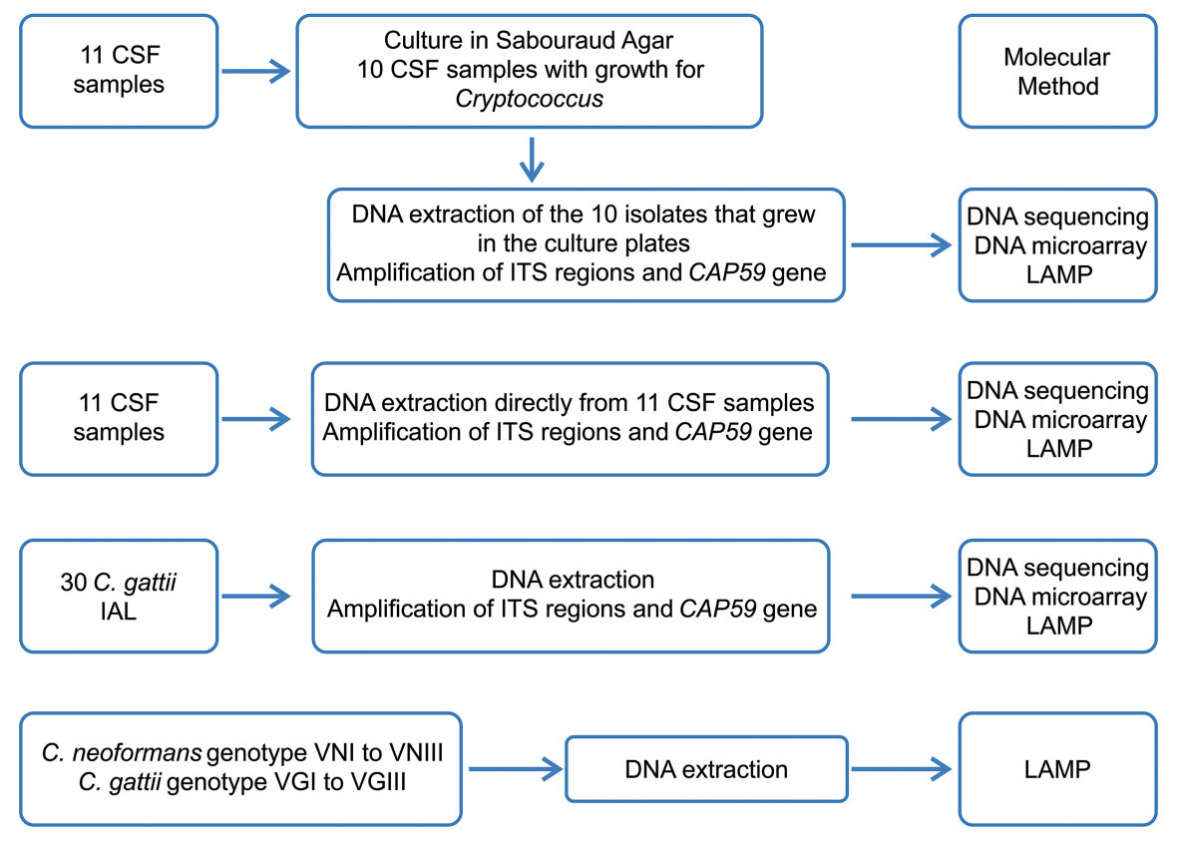
Flowchart of the molecular experiments using cerebral spinal fluid from patients with cryptococcal meningitis, *Cryptococcus gattii* isolates, and DNA samples of *Cryptococcus* spp. ITS: internal transcribed spacer; IAL: Instituto Adolfo Lutz; CSF: cerebral spinal fluid; LAMP: loop-mediated isothermal amplification.

The flowchart of experiments is shown in Figure 1.

### DNA extraction

Three protocols of CSF DNA extraction were previously tested and the protocol that used alkaline solution before the extraction by Qiagem DNA mini kit (Qiagen Sciences Inc., USA) had better performance (data not included). Two hundred microliters of CSF were added to 500 μL of alkaline solution (0.5 M NaOH + 0.05 M dehydrated trisodium citrate) and centrifuged at 16,000 *g* for 5 min at room temperature. The supernatant was removed and the precipitate was suspended in 500 μL of PBS followed by centrifugation at 16,000 *g* for 5 min at room temperature. The mixture was homogenized using MagNA Lyzer (Roche Applied Science, USA) at 5,000 *g* for 10 min. The precipitate was transferred to a 1.5-mL tube and proceeded with the DNA extraction using QIAmp DNA mini Kit, according to the manufacturer’s instructions. For DNA extraction from yeasts grown on culture plates, one loop from one pure colony was inserted in 500 μL of alkaline solution and followed the same protocol for CSF samples. The DNA extracted from CSF samples and from the grown yeasts on culture plates were quantified by NanoDrop™ 2000 Spectrophotometers (Thermo Fisher Scientific™, USA). The PCR products were amplified using specific primers for fungi targeting the internal transcribed spacer (ITS) region and *CAP59* gene.

To determine the detection threshold of *Cryptococcus* DNA in CSF, 10^7^ to 10^1^ yeast cells/mL of *C. neoformans* and *C. gattii* were artificially inoculated in CSF samples. For this experiment, we used CSFs with biochemical parameters (protein and glucose) and cytology within the normal range, and that cultured negative for bacteria, mycobacteria, and fungi. The DNA detection threshold was used for LAMP and DNA microarray assays.

### PCR reactions and DNA sequencing

PCR was performed using 5′-biotin-labeled fungus-specific universal primers; ITS1-bio (5′-TCCGTAGGTGAACCTGCGG-3′) and ITS4-bio (5′TCCTCCGCTTATTGATATGC-3′) (Sigma-Aldrich, USA) in order to amplify the entire ITS region and biotin labeling. For the gene amplification of *CAP59*, biotin-labeled specific primers *CAP59*-2R (5′-CRTTCATGAARACGACCG-3′) and *CAP59*-1F (5′-CKTGGTCCAACKCYGGCTG-3′) were used. The size of the amplified fragments varied from 426 to 930 bp depending on the fungal species. PCR was performed using PCR Master Mix (Promega^®^, USA). PCR reactions were incubated in Veriti 96-well Thermal Cycler (Applied Biosystems, USA) under the following conditions: 2 min for initial denaturation at 95°C, 40 cycles of DNA denaturation at 95°C for 30 s, primer annealing at 55°C for 30 s, elongation at 72°C for 1 min, and a final elongation step at 72°C for 5 min. For *CAP59* PCR reaction, the annealing temperature was 52°C. PCR products were verified by electrophoresis in a 2% agarose gel, 100 v, for 30 min. For DNA sequencing the PCR products were performed with the universal fungus-specific primers: ITS1 (5′-TCCGTAGGTGAACCTGCGG-3′) and ITS4 (5′TCCTCCGCTTATTGATATGC-3′), and *CAP59* Cap59-1F(5′-CKTGGTCCAACKCYGGCT-3′) and Cap59-2R(5′- CRTTCATGAARACGACCG-3′) (Sigma-Aldrich) followed by purification with ExoSAP-IT for PCR Product Clean-up (Affymetrix USB, USA), and the products were sequenced with the BigDye Terminator regent kit (Applied Biosystems), according to the manufacturer's protocols on an ABI Prism 3500 genetic analyzer (Applied Biosystems). The sequenced data were assembled and analyzed using BLAST (Basic Local Alignment Search Tool; http://blast.ncbi.nlm.nih.gov). DNA sequences were edited and assembled by Sequencher version 5.2.4 (Gene Codes Corp., USA). For identification, a homology search for the sequences ITS region and *CAP59* gene was done using the BLAST tool of the NCBI database (GenBank).

### Visual DNA microarray slides

The DNA microarray slide protocol used was as previously described by Sakai et al. (18) with modifications. Briefly, the oligonucleotide probes consisted of species/genus-specific nucleotide sequences with biotin-labeled poly(T) anchors at the end of each nucleotide (Sigma-Aldrich), which were designed based on the ITS sequences (ITS1 and ITS2) of the typed strains (GenBank database, American Type Culture Collection, CBS, and the Medical Mycology Research Center, Chiba University, Japan). This microarray platform covered 14 genera and 31 species of pathogenic fungi (Supplementary Figure S1A). A new platform, *CAP59* platform, was designed to better differentiate the two *Cryptococcus* species using the probes for *CAP59* gene regions (Supplementary Figure S1B). The previous platform was based on ITS regions resulting in cross-reaction between *C. neoformans* and *C. gattii.* The probe sequences were spotted onto polycarbonate slides (NGK Insulators LTD, Japan) with a KCS mini microarray printer (Kubota Comps Corporation, Japan). All slides had positive controls for fungi, where universal signals for fungi were enclosed in dotted line frame at the bottom right of each slide. After the hybridization was completed, the results were visible to the naked eye. This microarray assay could be done in up to 6 h.

### Loop-mediated isothermal amplification

LAMP-method was performed as described elsewhere with modifications (24). A set of six primers were used for amplification of *Cryptococcus neoformans*: Set3_*neoformans-*(F3: GGTCGGTCTGAGGATCATCA; B3: TCTGTCTCCTACTCTGCCAA; FIP: TCGAACTTCGGCGAGGTATTCGGAACATCTATGCGTACCCGC; BIP: CGAGCTTCGTGACAATGACGGACAAGTCGTCCACGCAAGG; LF: GGTCTGTTCAACCATCGTAT; LB: GAAGTCTTCGACTCGGT) and for *Cryptococcus gattii*: Set1_*gattii*-(F3:CCAGACAAAGGCGCTCTTG; B3: ATCGTTCATGAAGACGACCG; FIP: ACGGGTACGCATGGATGTTCGGATTTTCGATGCCCTCGCG; BIP: TCGAATACCTTGCCGAAGTCCGCCGTCATTGTCACGGAGTTC; LF: GATGATGATCCTGAGACCGACG; LB: AAACGCCGCCATGCTGC). The primer sets were designed for the *CAP59* gene using Primer Explorer V5 (https://primerexplorer.jp/e/). The amplification reaction of the assay was composed of 10 μL of each FIP and BIP primer at a concentration of 40 pmol, 10 μL of each F3 and B3 primer at 5 pmol concentration, 10 μL of LF and LB in a concentration of 20 pmol, 2 μL of extracted DNA, 100 μL of reaction mix (1 mL of 8 M betain, 500 μL of 165 mM MgCl_2_, 500 μL Tris-HCL, 500 μL KCL, 500 μL NHSO_4_, 500 μL 2% Tween 20, 350 μL 10 mM dNTP, and 100-300 μL dNTP 10 mM filtered water), 10 μL Bst DNA polymerase, and 30 μL filtered water. This mixture was incubated in a turbidimeter (Loopamp EXIA, Japan) at 63°C. Positive results were observed when the amplification curve appeared up to 60 min after starting the reaction. The turbidimeter of the LAMP reaction was analyzed in a real-time turbidimeter (Loopamp EXIA).

## Results

A total of 133 CSF samples were included. Eleven CSFs were positive for *Cryptococcus* from nine patients, 15 samples were positive for bacteria, and 107 CSF samples were negative for fungi, bacteria, and mycobacteria (Table 1). Six patients had meningitis due to *C. neoformans* and two patients due to *C. gattii*. The CSF samples were named as followed: P232 (patient 1; *C. neoformans*), P260 and P261 (patient 2; *C. neoformans*), P272 (patient 3; *C. gattii),* P273 and P274 (patient 4; *C. neoformans*), P275 (patient 5; *C. neoformans*), P276 (patient 6; *C. neoformans*), P283 (patient 7; *C. neoformans*), P284 (patient 8; *C. neoformans*) and P285 (patient 9; *C. gattii*) (Table 1). P272 CSF sample did not grow in culture, and the diagnosis was made by microscopy that showed fungal elements suggestive of *Cryptococcus* and by DNA sequencing of the PCR product. The DNA quantification of the 11 CSF samples varied from 4.2 to 11.2 ng/μL whereas the DNA obtained from *Cryptococcus* isolates varied from 11.9 to 65.4 ng/μL. For the artificially inoculated CSF samples, the limit of detection of the PCR reactions targeting ITS regions of *C. neoformans* and *C. gattii* was 10^2^ cells/mL in both species (data not shown).

### DNA sequencing of ITS regions and </emph>***CAP59* gene amplification**

The DNA sequencing of ITS regions and *CAP59* gene was made for all PCR amplification products to ensure that the resulting PCR amplification products were *Cryptococcus* and to confirm the species. The 15 CSFs positive for bacteria and the 107 negative CSF samples did not show any amplification for ITS regions and *CAP59* gene. The 30 *C. gattii* strains from the IAL collection analyzed by DNA sequencing presented 99 to 100% homology with the NCBI database. The sequencing of the ITS regions and *CAP59* gene of the DNA extracted directly from the 11 clinical samples and from their respective isolates grown on culture plates showed a percentage of homology ranging from 98 to 100% compared to the NCBI database. Our results suggested that *CAP59* gene amplification was a suitable target for sequencing *Cryptococcus* (Supplementary Table S1). The sequences were deposited in the NCBI data bank accession numbers (MK369690-MK369710).

### ITS and </emph>***CAP59* DNA microarray platforms**

**Figure 2:**
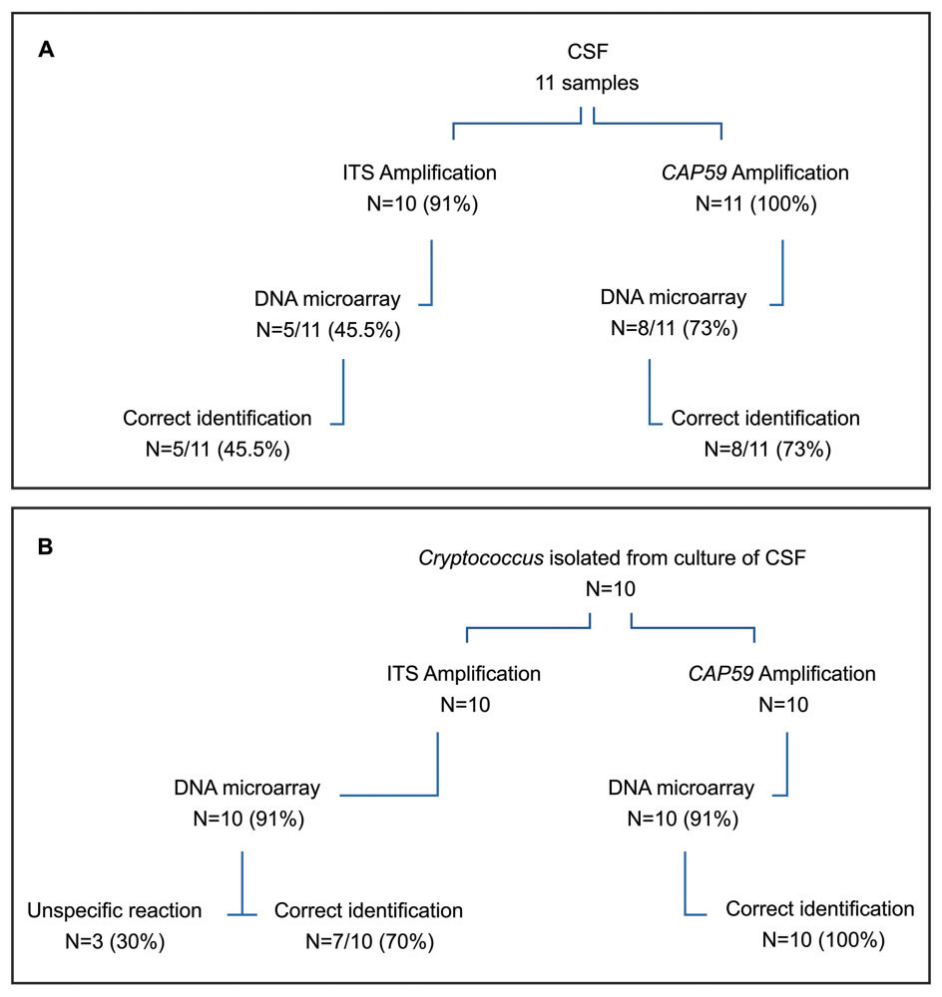
Results of the identification of ITS and *CAP59* DNA microarray platforms. Flowchart **A** shows the results of DNA extractions directly from clinical samples of cerebral spinal fluids (CSF). Flowchart **B** shows the results of DNA extractions obtained from *Cryptococcus* isolates grown in culture medium after CSF cultivation.

**Figure 3:**
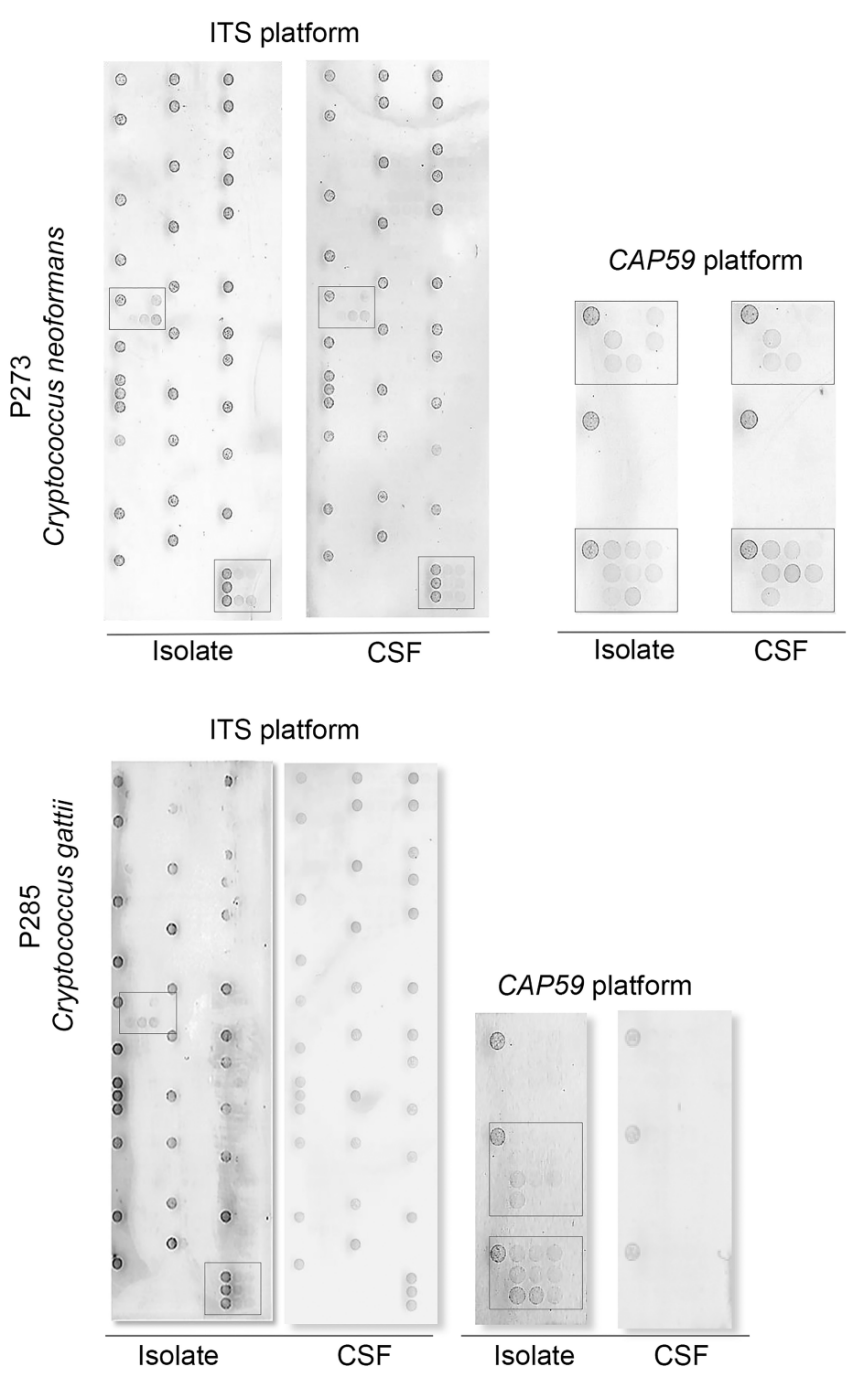
Representative results of *Cryptococcus* identification by DNA microarray platforms. P273 cerebral spinal fluid (CSF) sample and cultured isolate of *C. neoformans* tested by ITS and *CAP59* platforms showing positive results, and P285 CSF sample and cultured isolated of *C. gattii* tested by ITS and *CAP59* platforms showing positive results only for DNA extraction obtained from cultured isolates.

The microarray platforms were tested for the identification of species/genus-specific hybridization patterns. The original microarray platform was designed to target the ITS region; however, the probes showed cross-hybridization within the *Cryptococcus* genus because of their highly conserved sequence (18). To improve the discrimination of *Cryptococcus* species, the *CAP59* microarray platform was then developed. *CAP59* microarray was firstly tested for the 30 *C. gattii* isolates from the IAL collection and showed 100% concordant results with DNA sequencing. For the experiments using DNA extracted from CSFs, *CAP59* platform showed a better performance in the identification and discrimination of *Cryptococcus* species than the ITS platform. *CAP59* platform identified the species of 73% (n=8/11) CSF samples whereas ITS platform identified 45.5% (n=5/11) (Figures 2 and 3). Better results were observed when the DNA microarray was performed using DNA extracted from cultivated isolates from CSFs samples (Figure 2B). *CAP59* identified genus/species correctly in 100% and ITS in 70% of the *Cryptococcus* DNA.

### LAMP

**Figure 4:**
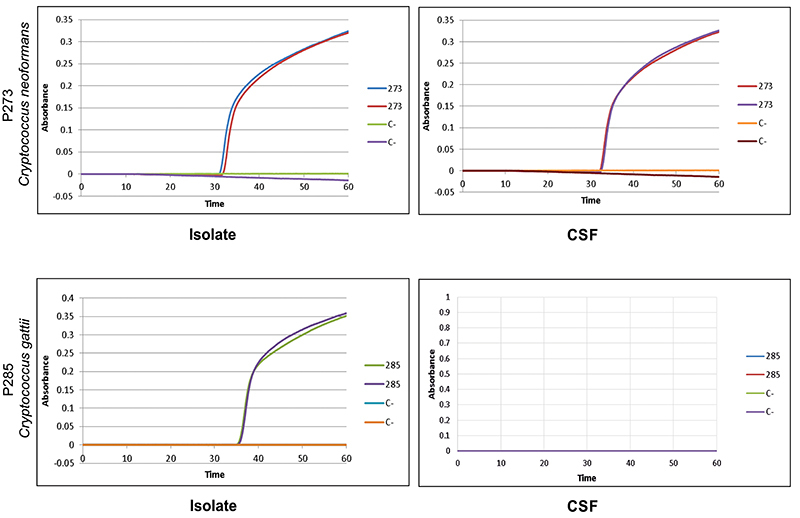
Representative loop-mediated isothermal amplification (LAMP) results of P273 cerebral spinal fluid clinical sample and culture (C). Isolate of *Cryptococcus neoformans* showed positive results after 30 min of reaction time. P285 showed a positive result after 35 min of reaction time only for DNA extracted from the clinical isolate of *Cryptococcus gattii.*

The primer set Set3_*neoformans* was firstly tested for the *C. neoformans* genotypes VNI, VNII, and VNIII, and the results are reported in Figure S2. The primer set Set1_*gattii* correctly identified the 30 isolates of *C. gattii* from IAL collection suggesting that this set of primer might be useful in the identification of cryptococcal DNA from CSF of patients with cryptococcal meningitis due to *C. gattii*. Set3_n*eoformans* and Set1_*gattii* identified all *C. neoformans* and *C. gattii* isolates, respectively (Figure 4), when the DNAs used in the reaction were extracted from the isolates cultivated on culture plates. The two sets of primers identified the nine *C. neoformans* isolates (P232, P260, P261, P273, P274, P275, P276, P283, P284) and the P285 *C. gattii* isolate. However, when the same set of primers, Set3 *neoformans* and Set1 *gattii*, were applied to amplify the DNA extracted from CSF, exponential curves were seen in 55.5% (n=5/9) of *C. neoformans* and none for the two *C. gattii* samples. The threshold of detection test performed with *C. neoformans* ATCC90113 showed that 10^4^ cells/mm^3^ were necessary to obtain a good exponential curve.

## Discussion

In our study, two molecular methodologies, DNA microarray and LAMP, were tested in the identification and discrimination of *Cryptococcus* species from the CSF of patients with cryptococcal meningitis, and from the *Cryptococcus* isolates of CSF samples that were seeded in culture medium.

Several studies with array platforms were designed to identify one or more fungal species in the same assay (13,18,30,31). The insertion of oligonucleotide sequences for the detection of various genera and fungi species in a single platform reduces the operational cost and allows the identification of several species of fungi in the same reaction and the observation of cross reactions. In our study, ITS and *CAP59* platforms identified and discriminated the genus *Cryptococcus* from both *Cryptococci* DNAs extracted from CSF and from the cultured isolates. ITS platform identified 45.5 and 70% and *CAP59*, 73 and 100% of the CSF samples and cultured isolates, respectively (Figure 2B). *CAP59* platform seemed to be more discriminatory than ITS platform for the identification of the genus *Cryptococcus* and the species *neoformans* and *gattii*. Our microarray DNA platform developed to identify *Cryptococcus* to species level using probes designed based on *CAP59* gene demonstrated 100% agreement in the identification of species of *C. gattii* when the DNA was extracted from clinical and environmental isolates, from the IAL collection. Although only 11 CSF samples were tested, these results might suggest that *CAP59* could be included as a target gene in microarray platforms for further studies with *Cryptococcus* (Figure 2). 

In our experiments, the amount of fungal DNA extracted from CSF was lower than the DNA obtained from the culture isolates, and the amount of DNA may had interfered with the microarray hybridization. For further conclusions, a larger number of clinical CSF samples from patients with cryptococcal meningitis will need to be tested in our platforms. O'Halloran et al. (32) failed to diagnose cryptococcal meningitis when CSF was studied by the approved commercialized FilmArray meningitis/encephalitis diagnostic panel (BioFire Diagnostic, USA). The authors suggested that patients with low fungal load in CSF were more likely to have a false negative test, emphasizing the importance of the amount of DNA in the molecular diagnostic tests.

LAMP tests were performed with two sets of primers that were designed based on the *CAP59* gene: Set3_*neoformans* and Set1_*gattii*. These two sets were initially tested in DNA from clinical and environmental isolates of *C. gattii* from the IAL collection (genotypes VGI, VGII, VGIII, VNI, VNII, and VNIII) resulting in the correct identification of all the tested isolates (Figure S2). The primers underwent several tests with different samples, and we obtained 100% agreement with the results of DNA sequencing, absence of exponential curves for other microorganisms, such as *Klebsiella* sp., *Candida albicans*, *Fusarium solani*, *Candida parapsilosis*, *Histoplasma capsulatum*, and for negative controls. Only one previous study by Lucas et al. (33) had performed LAMP based on the *CAP59* gene to identify the serotypes A, D, and B/C of *Cryptococcus* species complex, resulting in 83% agreement with a commercial serotyping kit.

Similarly to DNA microarray results, when testing CSF samples for LAMP assays, we had amplification in 55.5% of the CSF samples of patients with meningitis due to *C. neoformans* and no amplifications for the *C. gattii* CSF. Our results suggested that, for testing CSF samples, LAMP assay might need a higher number of *Cryptococcus* cells, for example, a larger volume of the clinical sample, to obtain a higher amount of DNA for the test. The mean amount of DNA obtained from the *Cryptococcus* grown in the culture media was significantly higher (P<0.001) (33.45 ng/μL) than the DNA obtained directly from CSF samples (6.85 ng/μL). However, the mean DNA quantification of CSF samples that resulted in a LAMP positive reaction (7.18 ng/μL) compared to the LAMP negative samples (6.46 ng/μL) was not significantly different (P=0.71) (data not shown). We observed that the specimen source played an important role in the results of DNA extraction. If the template was prepared from pure cultures, the amount of DNA was not a limiting factor. But working directly with clinical specimens such as CSF was challenging for many reasons. Low amounts of fungal cells can often be found in clinical specimens and host nucleic acids or proteins may occupy fungal targets, reducing the performance of the test. These problems might be overcome by optimizing the steps of precipitation and purification during DNA extraction to obtain sufficient amounts of DNA free of interferences.

In conclusion, DNA microarray and LAMP tested in this study based on ITS region and *CAP59* gene could be applied in clinical and epidemiological studies for the diagnosis of cryptococcosis. *CAP59* gene showed to be a promising target for future studies on molecular diagnostic tests. Moreover, they can be used as complementary tests to the microbiological diagnoses and antigen assays in the differentiation of *Cryptococcus* species in clinical practice.
